# Cases of usual ultrasound

**DOI:** 10.1186/1824-7288-41-S2-A33

**Published:** 2015-09-30

**Authors:** R Galiano

**Affiliations:** 1 UTIN, Azienda Ospedaliera “Pugliese Ciaccio”, Catanzaro 88100, Italy

## Background

Ultrasound (US) can be decisive for diagnosis, in some situations, in others the contribution of the US is only complementary and must be integrated with other diagnostic tests. Finally, in some cases, the ultrasound imaging does not provide solutions, but offers questions. The clinic, experience, and knowledge can find appropriate solutions.

## When an ultrasound is a diagnosis

**Figure 1:**
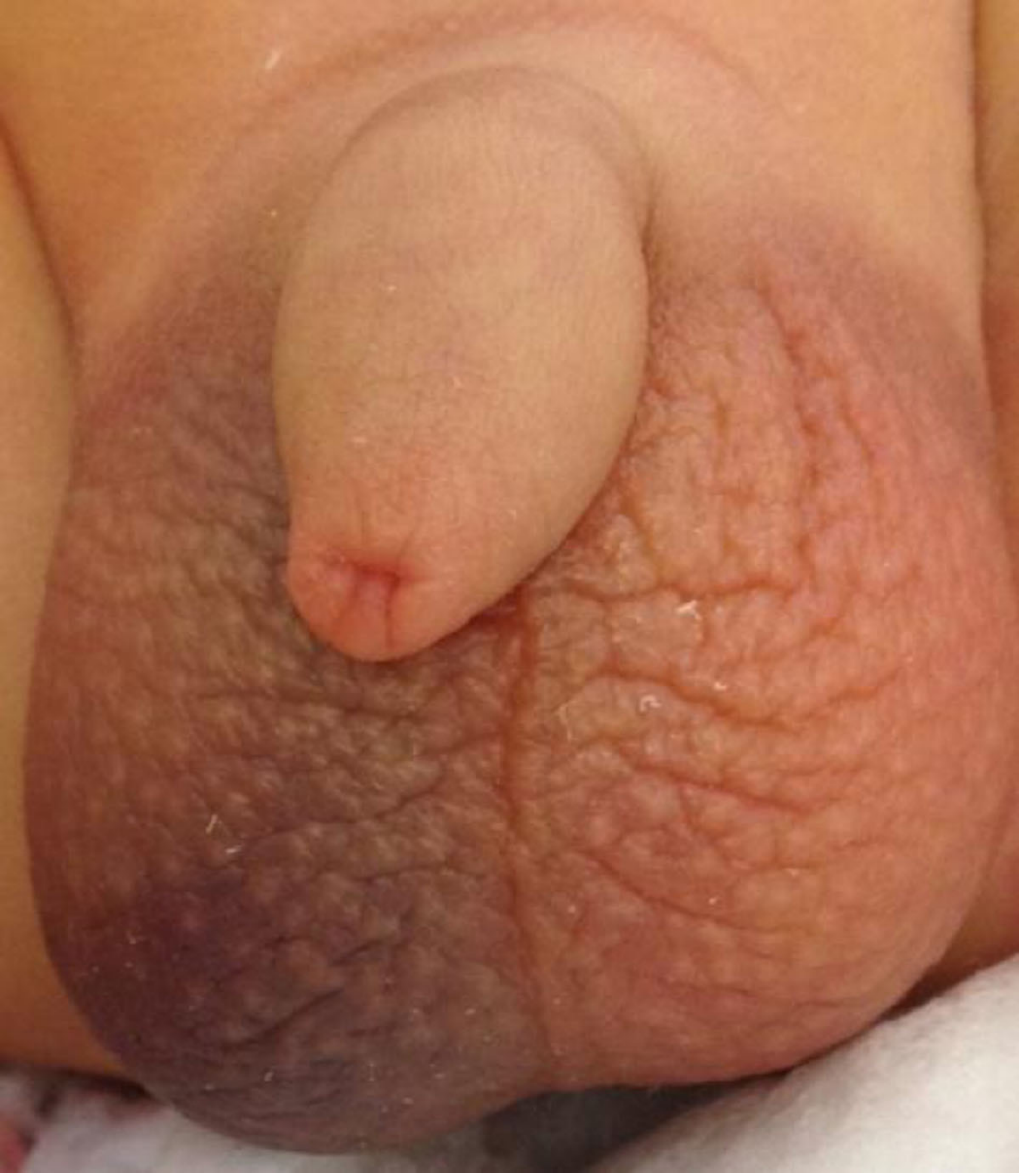
Hematoma of the right hemiscrotum.

**Figure 2:**
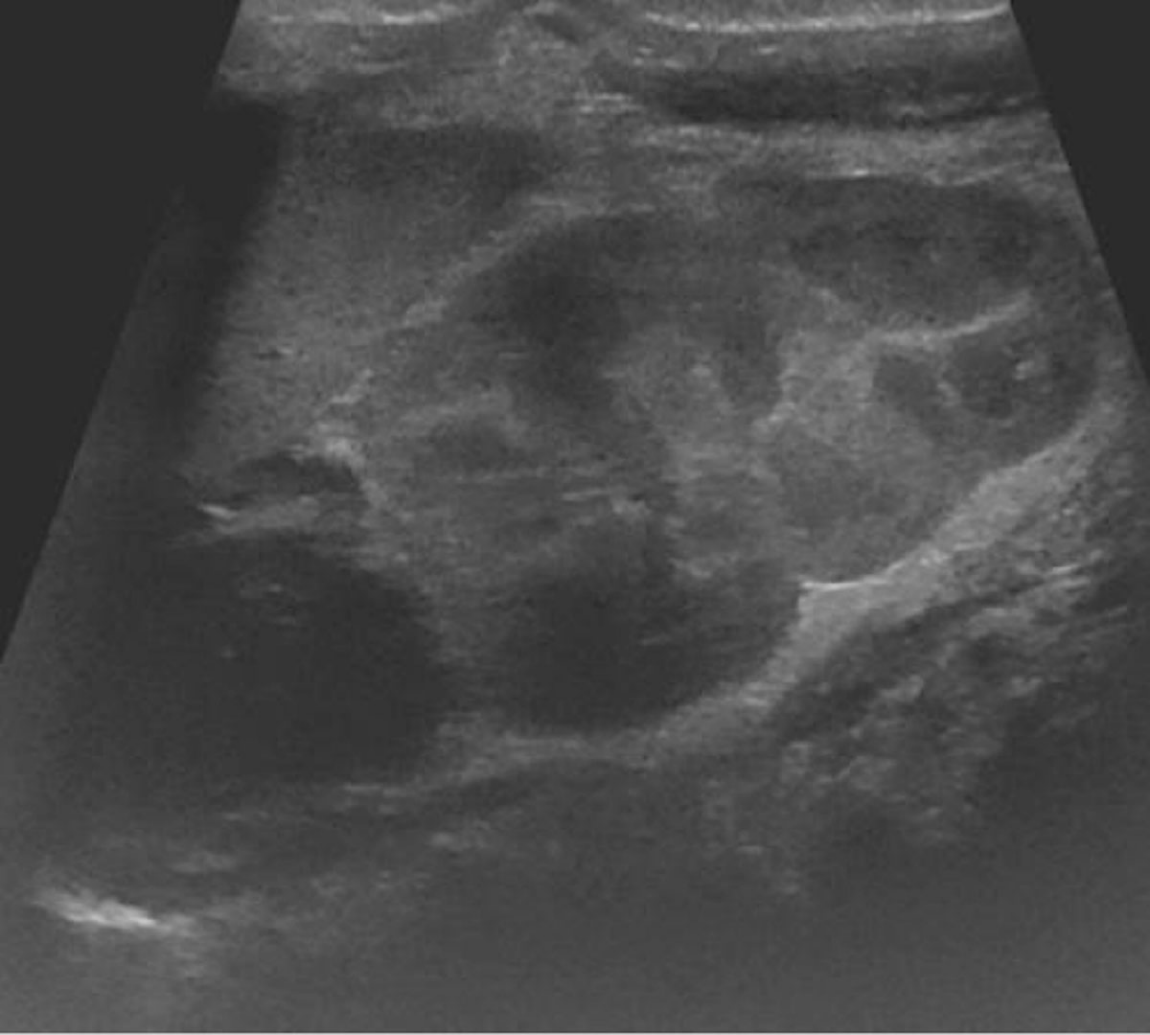
Complex mass upper pole of the right kidney

A male newborn of 3700 g., born after 40 weeks’ gestation via a vaginal dystotic delivery, Apgar 8 and 10 to 1'and 5', had a fracture of the right clavicle. After about 48 hours he was admitted to our department for jaundice and hematoma of the right hemiscrotum (Fig.1), with no pain, no other symptoms. Scrotal Doppler US showed a normal testicle size and shape, regularly vascularized, but modest collection of fluid, finely corpusculated and marked thickening of subcutaneous tissue. Abdomen US revealed a complex mass upper pole of the right kidney (2.8 cm), not vascularized, withinhomogeneous echogenicity (Fig.2). Ultrasonography had been sufficient to make the diagnosis of adrenal hemorrhage with hemorrhagic spreading at right hemiscrotum. Were not performed further investigations and a conservative treatment was chosen. Ultrasound monitoring showed progressive organization of the hematoma and complete resolution after about 40 days.

## When the ultrasound helps in diagnosis

**Figure 3:**
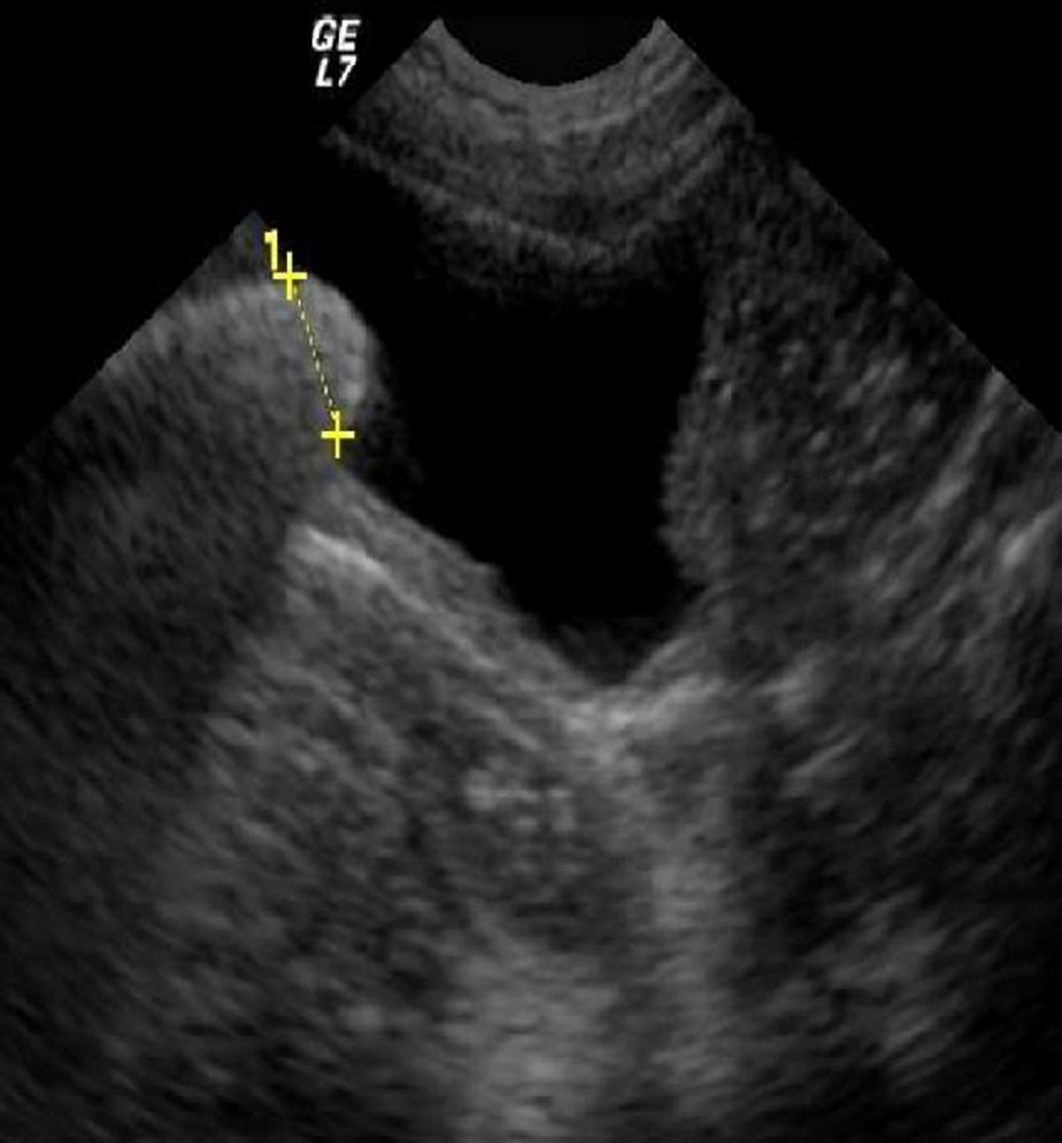
One large stone bladder

A boy, six years old, previously healthy, about 15 days ago was treated with oral antibiotics for UTI. An ultrasound is performed because urinary frequency, dysuria, urgency, gross hematuria reappeared. US revealed one large stone bladder (Fig.3); microscopy of the urine sediments showed cystine crystals. Stone, removed from the bladder endoscopically, were composed of cystine and calcium oxalate, when analysed.

## When the ultrasound raises clinical questions

Routine fetal ultrasound screening reports dilatation of the urinary tract in about 1-2% of all pregnancies, most of these are mild or moderate, limited to the renal pelvis, calyceal and ureter is not seen, the bladder is normal, renal parenchyma have normal thickness and appearance. These children do not have an obstructive pathology, nor a predisposition to UTI, and in most cases the dilatation is transient and has no pathological significance. Only clinical experience and up to date knowledge avoids these children to undergo invasive, painful and expensive imaging techniques, or to strenuous follow-up.

Written informed consent for publication of clinical details and clinical images was obtained from the parents of the patients

